# Supplementary material for: Prey killing without invasion by Bdellovibrio bacteriovorus defective for a MIDAS-family adhesin
Source: Nat Commun. 2024 Apr 9;15:3078. doi: 10.1038/s41467-024-47412-3 (PMC11003981; doi:10.1038/s41467-024-47412-3)
Supplement: Supplementary file 1 — Supplementary Information [file 41467_2024_47412_MOESM1_ESM.pdf]

Supplementary Information for: Prey killing without invasion by *Bdellovibrio bacteriovorus* defective for a MIDAS-family adhesin

Jess Tyson, Paul Radford, Carey Lambert, Rob Till, Simona G. Huwiler, Andrew L. Lovering*, R. Elizabeth Sockett*

Source Data and Supplementary Dataset1 Proteomics Data are provided as separate files See Data Availability section in main manuscript.

Supplementary Table 1. Pilot transcriptional experiment used to select MIDAS candidate genes for further study

Pilot comparative transcription of selected MIDAS protein genes and known predatory protein genes (for DacBs and LDTPs that modify prey cells[1,2]), tested upon prey-attachment by prey/host-independent (HI) strains (1291) of Δ*bd1291* deletion mutant (a *B. bacteriovorus* HI strain with prolonged prey attachment, without invasion) compared to wild type prey/host-independent strains (HID) which invade prey albeit at a variety of rates due to their diverse cell lengths, and partial predatory differentiation which are products of HI growth. Comparison of these strains did not constitute an endpoint experiment to identify important predatory proteins definitively, but instead gave testable candidates which were then mutated and fluorescently tagged and monitored microscopically to define their roles in predation.

Comparative data for 1291/HID shown in columns 4-7 with the transcriptional change strain Δ*bd1291*/HID wild type, in presence of prey in bold/shaded in column 3. (Raw data for each of two biological replicates of the HID strains are shown in columns 8-13 and for the Δ*bd1291* mutant strains in 14-19). These pilot data (although not full transcriptional study) allowed us to propose the hypothesis that Bd0875 was suitable for testing as a potential adhesin important during predation, which we then did by mutagenesis and gene tagging and predation studies.

**Gene number**	Known function / motif	**log2 of fold change: 1291/HID Expression**	fold change: 1291/HID Expression	fold change: 1291/HID RPKM	pValue HIDs plus S17 vs 1291s plus S17	qValue HIDs plus S17 vs 1291s plus S17	Raw Counts HIDs plus S17 Replicate 1	Raw Counts HIDs plus S17 Replicate 2	Normalized Counts HIDs plus S17 Replicate 1	Normalized Counts HIDs plus S17 Replicate 2	RPKM HIDs plus S17	Expression HIDs plus S17	Raw Counts 1291s plus S17 Replicate 1	Raw Counts 1291s plus S17 Replicate 2	Normalized Counts 1291s plus S17 Replicate 1	Normalized Counts 1291s plus S17 Replicate 2	RPKM 1291s plus S17	Expression 1291s plus S17
bd0767	MIDAS-motif containing	**-1.58**	0.333	0.236	1.47E-04	1.60E-03	3463	2664	197663	107910	199	93	867	696	48761	54832	47	31
bd0875	MIDAS-motif containing	**-4.76**	0.037	0.025	0.00E+00	0.00E+00	7519	12279	429149	497358	924	407	293	245	16490	19345	23	15
bd3132	MIDAS-motif containing	**-1.08**	0.474	0.310	1.21E-02	9.33E-02	1792	2130	102291	86310	126	57	566	735	31850	57948	39	27
bd0816	DacB rounds prey	**-5.54**	0.022	0.014	0.00E+00	0.00E+00	1947	3327	111125	134777	213	93	37	46	2131	3697	3	2
bd3459	DacB rounds prey	**-4.01**	0.062	0.042	4.31E-209	1.10E-207	4396	7698	250927	311812	478	209	304	247	17105	19484	20	13
bd0886	LDTPase reinforces dAla	**-2.64**	0.160	0.100	1.50E-25	2.68E-24	2203	4166	125728	168747	231	100	334	369	18820	29131	23	16
bd1176	LDTPase reinforces dAla	**-5.11**	0.029	0.018	0.00E+00	0.00E+00	9062	13903	517214	563130	780	345	218	255	12273	20148	14	10

For this pilot experiment, HI strains were grown overnight in PY broth at 30°C with shaking at 200 rpm and *E. coli* S17-1 was grown in YT broth at 37°C with shaking at 200 rpm. Strains HID13 and HID22 (wild-type), two isolates of strain Δ*bd1291* (stalled prey entry) and prey *E. coli* S17-1 were backdiluted to OD_{600} of 1.0 in Ca/HEPES buffer before mixing at a ratio of 1:1 in a final volume of 100 ml. Controls of *E. coli* S17-1 prey only were also performed by mixing with Ca/HEPES buffer instead of *B. bacteriovorus*. These samples were incubated for 15 mins at 30°C with shaking at 200 rpm before 10 ml samples being removed into 1% phenol 5% ethanol (v/v) and being processed for total RNA extraction and QC using the Agilent 6000 machine and Nano Chip kit according to the manufacturer's instructions [3,4]. Ten micrograms of total RNA were sent to Vertis Biotechnologie AG for their standard pipeline of rRNA depletion and sequencing via Illumina HiSeq. FastQ files were used with the Rockhopper app [5] using default parameters (in verbose mode) and treating the two wild type strains and two mutant strains as replicates. The assembled table below originates from the Rockhopper output as well as manual calculation of fold change values and log2 thereof in Excel (Microsoft).

Supplementary Table 2. MIDAS protein-encoding gene transcription patterns at 30 minutes after predatorily grown *B. bacteriovorus* HD100 mixing with *E. coli* S17-1 prey, during invasion. Transcription data interrogated for fold change on entry of predator Bdellovibrio into prey bdelloplast are reported here, for just those genes to highlight their different levels of expression, they are extracted from published data [3]. Those data are at GEO Gene expression Omnibus GSE9269. [https://www.ncbi.nlm.nih.gov/geo/query/acc.cgi?acc=GSE9269]

Fold-Change upon entry to bdelloplast results from comparing transcriptome at 30 minutes of predatory growth versus attack phase.

T-test was a paired, two-tailed test for each comparative experimental condition. For further details please refer to Figure S1 in Lambert et al., 2010 [3].

Gene Number	Gene Name (predicted protein function)	Fold-Change upon entry to bdelloplast	T-test
Bd0875	hypothetical protein	3.07	4.93E-02
Bd0767	leucyl-rRNA synthetase	0.68	2.80E-02
Bd1483	hypothetical protein	0.73	1.34E-01
Bd3132	hypothetical protein	0.73	5.88E-02

Supplementary Table 3.

a. Primers used in this study.

Primer	Sequence (5' to 3')	Purpose
1291KOgibForF	TAAAACGACGGCCAGTGCCATGGCTTGCTCTTCTGTTTTC	Deletion of *bd1291*
1291KOgibForR	TTCGGTGTTGATCCATGAAAAACCTATGGG	
1291KOgibRevF	TTTCATGGATCAACACCGAACAAGATCC	
1291KOgibRevR	CAGCTATGACCATGATTACGACCACCAGACGTGGTGAAC	
875mCh_v2_UP_F	CGTTGTAAAACGACGGCCAGTGCCATTGCGTGTTTTAGCTGTACC	Bd0875-mCherry C terminal fusion for single crossover into HD100 WT strain
875mCh_v2_UP_R	CCTTGCTCACCATGTCCACGCGTTTGATTTG	
875mCh_v2_DN_F	CAAACGCGTGGACATGGTGAGCAAGGGCGAG	
875mCh_v2_DN_R	GGAAACAGCTATGACCATGATTACGTTACTTGTACAGCTCGTCCATG	
Bd875_D66A_F	TATAGTAAATGCCAACTCTGCAT	Bd0875 D66A point mutant
Bd875_D66A_R	AGGATGTCGACCTTACCGCG	
Bd875_E213A_F	CCTGGCGGACGCCGATGAAAGATC	Bd0875 E213A point mutant
Bd875_E213A_R	AAGATCACCGCCAGGTGAGC	
Bd875_Y349A_F	CGCCGGTGGGTGCCAATCTGGTGG	Bd0875 Y349A point mutant
Bd875_Y349A_R	AACCGGACACAGTCACAATC	
Bd767UP_F	cgttgtaaaacgacggccagtgccaCGCTGGTCTGTTGACCATC	Deletion of *bd0767*
Bd767UP_R	tagcttacttaatCTTCATACCTTCTCCCTGCTTC	
Bd767DN_F	agaaggtatgaagATTAAGTAAGCTACGGTTGGTGAAAAGG	
Bd767DN_R	ggaaacagctatgaccatgattacgATGGCGTGGAAAACCCGC	
Bd875UP_F	cgttgtaaaacgacggccagtgccaCTGGGAGTTGCTTTTTTATTC	Deletion of *bd0875*
Bd875UP_R	attgttagtctaaAGACACACCTCTCTCAACC	

Bd875DN_F	gagaggtgtgtctTTAGACTAACAATTTATTTTAGAGCG	
Bd875DN_R	ggaaacagctatgaccatgattacgTCCAAAAAGCTCTTTGTTTTAG	
Bd1483KO_UP_F	cgttgtaaaacgacggccagtgccaACGCCTTGAACGTGCCTC	Deletion of *bd1483*
Bd1483KO_UP_R	acagtttaatcgaTTTCATACGGTTATGCCCTCC	
Bd1483KO_DN_F	taaccgtatgaaaTCGATTAAACTGTAAAAGGATG	
Bd1483KO_DN_R	ggaaacagctatgaccatgattacgACCCATGAACTCATCCAC	
Bd3132UP_F	cgttgtaaaacgacggccagtgccaCGACTGGCTCCGGTGATC	Deletion of *bd3132*
Bd3132UP_R	cgtcttagaagacACTCATTGTGGAATATCAGGGG	
Bd3132DN_F	ttccacaatgagtGTCTTCTAAGACGCAGAGAG	
Bd3132DN_R	ggaaacagctatgaccatgattacgAGCCACAGTTTCATGATG	
Bd875UP_F	cgttgtaaaacgacggccagtgccaCTGGGAGTTGCTTTTTTATTC	Complementation of *bd0875*
Bd875DN_R	ggaaacagctatgaccatgattacgTCCAAAAAGCTCTTTGTTTTAG	

b. Plasmids used in this research study.

Plasmid	Description	Source
pK18*mobsacB*	Suicide vector (kanR, *lacZ*a, *sacB*) used for crossovers into the *B. bacteriovorus* genome	Schäfer *et al.* 1994 [6]
pAKF56-mCherry	Template for *mCherry* gene. Cloning vector for Bd0635mCherry	Fenton *et al.* 2010 [7]
p0064-mCh_SXO	Full length Bd0064-mCherry fusion (single-crossover	Willis *et al.* 2016 [8]
pDelta1291	Upstream and downstream fragments of *bd1291* gene for unmarked gene deletion	This study
p0875-mCh SXO	Full length Bd0875-mCherry fusion (single-crossover	This study
p0875_comp	Bd0875 from strain HD100 for double-crossover complementation	This study

p0875_D66A_comp	Bd0875 from p0875_comp with D66A point mutation for double-crossover complementation	This study
p0875_E213A_comp	Bd0875 from p0875_comp with E213A point mutation for double-crossover complementation	This study
p0875_Y349A_comp	Bd0875 from p0875_comp with Y349A point mutation for double-crossover complementation	This study
pDelta0767	Upstream and downstream fragments of *bd0767* gene for unmarked gene deletion	This study
pDelta0875	Upstream and downstream fragments of *bd0875* gene for unmarked gene deletion	This study
pDelta1483	Upstream and downstream fragments of *bd1483* gene for unmarked gene deletion	This study
pDelta3132	Upstream and downstream fragments of *bd3132* gene for unmarked gene deletion	This study

c. Strains used in this research study.

**Strains**	**Description**	**Source**
E. coli DH5a	*E. coli* cloning strain (*fhuA2Δ(argF-lacZ)U169 phoA glnV44 Φ80Δ(lacZ)M15 gyrA96 recA1 relA1*)	New England Biolabs (C2987)
E. coli S17-1	*E. coli* strain (thi, pro, hsdR-, hsdM+, recA; integrated plasmid RP4- Tc::Mu-Kn::tn)	Hanahan D., 1983 [9]
E. coli S17-1: pZMR100	*E. coli* strain containing the plasmid pZMR100 (kanR)	Rogers M, *et al.*, 1986 [10]
B. bacteriovorus HD100	*B. bacteriovorus* Type strain, genome-sequenced, wild-type	Rendulic S, *et al.*, 2004 [11]

B. bacteriovorus HI *Δbd1291*	Host-independent strain of *B. bacteriovorus* containing an in-frame unmarked deletion of *bd1291*	This study
B. bacteriovorus HD100 *bd*0064mCeru_DXO	HD100 containing a double-crossover, full length Bd0064- mCerulean fusion	Caulton SG *et al.*2024[12]
B. bacteriovorus HD100 *bd2740*mCh_SXO	HD100 containing a single-crossover, full length Bd2740-mCherry fusion expressed in attack phase (positive control).	Caulton SG *et al.*2024[12]
B. bacteriovorus HD100 *bd*0875mCh_SXO	HD100 containing a single-crossover, full length Bd0875-mCherry fusion	This study
B. bacteriovorus HD100 *Δbd0767*	*B. bacteriovorus* containing an in-frame unmarked deletion of *bd0767*	This study
B. bacteriovorus HD100 *Δbd0875*	*B. bacteriovorus* containing an in-frame unmarked deletion of *bd0875*	This study
B. bacteriovorus HD100 *Δbd1483*	*B. bacteriovorus* containing an in-frame unmarked deletion of *bd1483*	This study
B. bacteriovorus HD100 *Δbd3132*	*B. bacteriovorus* containing an in-frame unmarked deletion of *bd3132*	This study
B. bacteriovorus HD100 *Δbd0767* plus *Δbd0875* plus *Δbd3132*	*B. bacteriovorus* containing in-frame unmarked deletions of *bd0767* plus *bd0875* plus *bd3132*	This study
B. bacteriovorus HD100 *Δbd0875* complemented with *bd0875*	*B. bacteriovorus* containing an in-frame unmarked deletion of *bd0875* and complemented with an in-frame double crossover bd0875 from strain HD100	This study
B. bacteriovorus HD100*Δbd0875* complemented with *bd0875* D66A	*B. bacteriovorus* containing an in-frame unmarked deletion of *bd0875* and complemented with an in-frame double crossover Bd0875 D66A point mutant	This study
B. bacteriovorus HD100 *Δbd0875* complemented with *bd0875* E213A	*B. bacteriovorus* containing an in-frame unmarked deletion of *bd0875* and complemented with an in-frame double crossover Bd0875 E213A point mutant	This study

B. bacteriovorus HD100 *Δbd0875* complemented with *bd0875* Y349A	*B. bacteriovorus* containing an in-frame unmarked deletion of *bd0875* and complemented with an in-frame double crossover Bd0875 Y349A point mutant	This study
B. bacteriovorus HD100 *Δbd0767* plus *Δbd0875* plus *Δbd3132* complemented with *bd0875*	*B. bacteriovorus* containing in-frame unmarked deletions of *bd0767* plus *bd0875* plus *bd3132* and complemented with an in-frame double crossover bd0875 from strain HD100	This study

Supplementary Table 4. Measurement using MicrobeJ[13] of dimensions of 10% dead empty bdelloplasts versus 90% regular wild type-like invaded bdelloplasts, each caused by *Δbd0875* mutant predator invasion of *E. coli* S17-1 prey. Data derived from three biological repeats. NS = non-significant.

	**Overall data (3 biological repeats)**	**Dead empty bdelloplast (n=157)**	**Regular bdelloplast (n=735)**	**p value**	**p value summary**
Area (μm^2)	Mean	0.917	1.286	0.0006	***
	Std. Deviation	0.026	0.059		
Circularity (A.U)	Mean	0.989	0.986	0.1000	NS
	Std. Deviation	0.001	0.003		
Length (µm)	Mean	1.110	1.336	0.0003	***
	Std. Deviation	0.018	0.029		
Roundness	Mean	0.909	0.893	0.1242	NS
	Std. Deviation	0.012	0.007		
Width (µm)	Mean	1.007	1.172	0.0011	**
	Std. Deviation	0.009	0.033		

Supplementary Table 5.

Percentage of dead empty bdelloplasts at 120 mins post mixing of predator and prey: MIDAS deletion mutant and complemented strains. Tables **a.** (Mean and standard deviation), **b.** (p values from one-way ANOVA analysis take into account multiple comparisons of all strains tested against each other) and **c.** p values from two-tailed unpaired *t*-test), **d.** (number of bdelloplasts counted in total and per repeat) relate to data shown in Fig. 1c. Tables **e.** (Mean and standard deviation), **f.** (p values from one-way ANOVA analysis taken into account multiple comparisons of all strains tested against each other) and **g.** (number of bdelloplasts counted in total and per repeat) relate to data shown in Fig. 3b. Tables **g.** (Mean and standard deviation), **h.** (p values from one-way ANOVA analysis taken into account multiple comparisons of all strains tested against each other) and **i** (number of bdelloplasts counted in total and per repeat) relate to data shown in Fig. 3c. Ns = non-significant. [a]Analysis of *Δbd1483* was performed alongside a *B. bacteriovorus* HD100 control but in a separate experiment to analysis of *Δbd0767, Δbd0875, Δbd3132 and Δbd0767Δbd0875Δbd3132* which had a separate HD100 control.

a.

**Strain**	**mean and SD**
HD100	0.2 % +/- 0.2 %
ΔBd0767	0.2 % +/- 0.3 %
ΔBd0875	10.1 % +/- 2.9 %
ΔBd3132	0.3 % +/- 0.4 %
ΔBd0767ΔBd0875ΔBd3132	11.7 % +/- 4.3 %
HD100[a]	0.1% +/- 0.2%
ΔBd1483[a]	0.0% +/- 0.0%

b.

**Dunnett's multiple comparisons test**	**Summary**	**Adjusted P Value**
HD100 vs. ΔBd0767	ns	>0.9999
HD100 vs. ΔBd0875	**	0.0014
HD100 vs. ΔBd3132	ns	>0.9999
HD100 vs. ΔBd0767ΔBd0875ΔBd3132	***	0.0004

Tukey's multiple comparisons test	Summary	Adjusted P Value
HD100 vs. ΔBd0767	Ns	>0.9999
HD100 vs. ΔBd0875	**	0.0029
HD100 vs. ΔBd3132	Ns	>0.9999
HD100 vs. ΔBd0767ΔBd0875ΔBd3132	***	0.0009
ΔBd0767 vs. ΔBd0875	**	0.0027
ΔBd0767 vs. ΔBd3132	Ns	>0.9999
ΔBd0767 vs. ΔBd0767ΔBd0875ΔBd3132	***	0.0009
ΔBd0875 vs. ΔBd3132	**	0.0031
ΔBd0875 vs. ΔBd0767ΔBd0875ΔBd3132	Ns	0.9041
ΔBd3132 vs. ΔBd0767ΔBd0875ΔBd3132	***	0.001

c.

Two-tailed t test	Summary	P Value
HD100 vs ΔBd1483	ns	0.1242

d.

Strain	n (Expt 1)	n (Expt 2)	n (Expt 3)	Total n
HD100	553	370	212	1135
ΔBd0767	579	378	224	1181
ΔBd0875	525	317	279	1121
ΔBd3132	576	392	255	1223
ΔBd0767ΔBd0875ΔBd3132	570	344	248	1162
HD100[a]	303	294	405	1002
ΔBd1483[a]	355	359	376	1090

e.

Strain	mean and SD
HD100	0.6% +/- 0.3%
ΔBd0875	9.0% +/- 0.9%
ΔBd0767ΔBd0875ΔBd3132	8.2% +/- 1.6%
ΔBd0875:Bd0875	0.6% +/- 0.3%
ΔBd0767ΔBd0875ΔBd3132:Bd0875	0.5% +/- 0.2%

f.

Tukey's multiple comparisons test	Summary	Adjusted P Value
HD100 vs. ΔBd0875	****	<0.0001
HD100 vs. ΔBd0767ΔBd0875ΔBd3132	****	<0.0001
HD100 vs. ΔBd0875:Bd0875	Ns	>0.9999
HD100 vs. ΔBd0767ΔBd0875ΔBd3132:Bd0875	Ns	>0.9999
ΔBd0875 vs. ΔBd0767ΔBd0875ΔBd3132	Ns	0.8249
ΔBd0875 vs. ΔBd0875:Bd0875	****	<0.0001
ΔBd0875 vs. ΔBd0767ΔBd0875ΔBd3132:Bd0875	****	<0.0001
ΔBd0767ΔBd0875ΔBd3132 vs. ΔBd0875:Bd0875	****	<0.0001
ΔBd0767ΔBd0875ΔBd3132 vs. ΔBd0767ΔBd0875ΔBd3132:Bd0875	****	<0.0001
ΔBd0875:Bd0875 vs. ΔBd0767ΔBd0875ΔBd3132:Bd0875	Ns	>0.9999

g.

Strain	n (Expt 1)	n (Expt 2)	n (Expt 3)	Total n
HD100	629	223	329	1181
∆Bd875	583	327	241	1151
∆Bd767∆Bd875∆Bd3132	615	392	347	1354
∆Bd875:Bd875	517	306	313	1136
∆Bd767∆Bd875∆Bd3132:Bd0875	407	309	374	1090

h.

Strain	mean and SD
HD100	0.1% +/- 0.2%
∆Bd0875	9.3% +/- 1.7%
∆Bd0875:Bd0875	0.2% +/- 0.3%
∆Bd0875:Bd0875 D66A	7.5% +/- 1.2%
∆Bd0875:Bd0875 E213A	5.6% +/- 1.8%
∆Bd0875:Bd0875 Y349A	0.2% +/- 0.3%

i.

Tukey's multiple comparisons test	Summary	Adjusted P Value
HD100 vs. ΔBd0875	****	<0.0001
HD100 vs. ΔBd0875:Bd0875	Ns	>0.9999

HD100 vs. ΔBd0875:Bd0875 D66A	****	<0.0001
HD100 vs. ΔBd0875:Bd0875 E213A	***	0.0009
HD100 vs. ΔBd0875:Bd0875 Y349A	Ns	>0.9999
ΔBd0875 vs. ΔBd0875:Bd0875	****	<0.0001
ΔBd0875 vs. ΔBd0875:Bd0875 D66A	Ns	0.4338
ΔBd0875 vs. ΔBd0875:Bd0875 E213A	*	0.0172
ΔBd0875 vs. ΔBd0875:Bd0875 Y349A	****	<0.0001
ΔBd0875:Bd0875 vs. ΔBd0875:Bd0875 D66A	****	<0.0001
ΔBd0875:Bd0875 vs. ΔBd0875:Bd0875 E213A	**	0.0011
ΔBd0875:Bd0875 vs. ΔBd0875:Bd0875 Y349A	Ns	>0.9999
ΔBd0875:Bd0875 D66A vs. ΔBd0875:Bd0875 E213A	Ns	0.3636
ΔBd0875:Bd0875 D66A vs. ΔBd0875:Bd0875 Y349A	****	<0.0001
ΔBd0875:Bd0875 E213A vs. ΔBd0875:Bd0875 Y349A	**	0.001

j.

**Strain**	**n (Expt 1)**	**n (Expt 2)**	**n (Expt 3)**	**Total n**
HD100	303	294	405	1002
ΔBd0875	350	349	315	1014
ΔBd0875:Bd0875	426	398	363	1187
ΔBd0875:Bd0875 D66A	308	351	415	1074
ΔBd0875:Bd0875 E213A	408	319	346	1073
ΔBd0875:Bd0875 Y349A	392	410	339	1141

Supplementary Table 6. *B. bacteriovorus* proteomics data from Supplementary Dataset 1. filtered through WT gene expression data inside prey at 30 minutes[3].(Original published gene expression data available at GEO Gene expression Omnibus GSE9269. [https://www.ncbi.nlm.nih.gov/geo/query/acc.cgi?acc=GSE9269] **).**The list of gene name Bd numbers from the proteomics list (Supplementary Dataset 1) was compared to the list of genes in the predatorily expressed grouping named predatosome (upregulated during 30 minutes predation but not upregulated in HI growth; [3]) using the Python package Pandas (https://pandas.pydata.org/pandas-docs/stable/getting_started/overview.html) and the merge command with inner specified. PSMs = peptide-spectrum matches.

Synonym	Gene Name	Fold-Change upon entry to bdelloplast	T-test	Fold-Change upon HI growth	T-test	Accession	# AAs	MW [kDa]	calc. pI	Description	Coverage	Score	# Unique Peptides	# Peptides	# PSMs	Area
Bd0173	hypothetical protein	1.58	0.00714	0.82	0.00354	Q6MRB7	921	103	8.84	Uncharacterized protein OS	2.28	0	2	2	2	5.50E+07
Bd0401	hypothetical protein	4.46	0.00252	4.6	0.338	Q6MQQ9	725	77.7	8.53	Uncharacterized protein OS	2.07	2.22	1	1	1	7.56E+08
Bd0697	putative actin-binding protein	1.63	0.00389	0.63	0.0011	Q6MPZ1	170	19.1	10.05	Putative actin-binding protein OS	5.29	0	1	1	1	1.26E+08
Bd0886	hypothetical protein	5.12	0.00494	2.59	0.0649	Q6MPG8	486	53.5	8.98	SH3b domain-containing protein OS (LD transpeptidase[2])	1.85	1.83	1	1	1	5.70E+07
Bd0993	putative secreted protein	2.46	0.0268	4.67	0.308	Q6MP71	529	60.2	8.7	Putative secreted protein OS	7.75	5.2	3	3	3	1.16E+08
Bd1012	hypothetical protein	1.83	0.0363	2.64	0.0659	Q6MP53	1336	148.8	8.46	Uncharacterized protein OS	4.04	3.75	4	4	4	1.16E+08
Bd1044	hypothetical protein	2.44	0.00201	3.46	0.018	Q6MP24	481	53.2	8.47	BD_b_sandwich domain-containing protein OS	5.41	0	1	1	1	5.29E+07
Bd1045	hypothetical protein	3.38	0.00623	3.93	0.0872	Q6MP23	514	55.7	8.69	BD_b_sandwich domain-containing protein OS	2.14	0	1	1	1	4.43E+08
Bd1047	hypothetical protein	5.22	4.92E-05	4.51	0.153	Q6MP21	321	35	9.61	Uncharacterized protein OS	29.91	18.89	7	7	9	2.52E+09
Bd1176	hypothetical protein	6.25	0.0106	7.1	0.0148	Q6MNR2	520	57.4	8.59	Uncharacterized protein OS (LD Transpeptidase[2])	2.31	0	1	1	1	3.52E+08
Bd1399	hypothetical protein	1.3	0.0971	1.15	0.345	Q6MN62	185	19.7	8.43	Uncharacterized protein OS	4.86	0	1	1	1	3.32E+08

Synonym	Gene Name	Fold-Change upon entry to bdelloplast	T-test	Fold-Change upon HI growth	T-test	Accession	# AAs	MW [kDa]	calc. pI	Description	Coverage	Score	# Unique Peptides	# Peptides	# PSMs	Area
Bd1541	putative protease	2.22	0.000174	1.2	0.42	Q6MMT2	299	31.5	6.89	Putative protease OS	5.02	0	1	1	1	
Bd1542	hypothetical protein	6.48	0.000302	2.2	0.297	Q6MMT1	398	44.3	4.86	Uncharacterized protein OS	3.77	0	1	1	1	
Bd1904	hypothetical protein	13.92	0.00118	0.65	0.000943	Q6MLV0	181	20.6	9.38	Uncharacterized protein OS	7.73	2.44	1	1	1	4.56E+08
Bd2005	hypothetical protein	3.82	6.44E-05	0.88	0.0507	Q6MLK3	325	36	5.07	Uncharacterized protein OS	5.85	0	2	2	2	7.00E+08
Bd2195	hypothetical protein	3.99	0.000287	0.58	0.000279	Q6ML27	157	17	5.4	Uncharacterized protein OS	9.55	2.12	1	1	1	1.47E+08
Bd2382	hypothetical protein	1.6	0.0321	1.13	0.0665	Q6MKK6	1098	122.1	8.46	Uncharacterized protein OS	1.28	0	1	1	1	1.59E+07
Bd2430	hypothetical protein	6.11	0.0201	1.14	0.0719	Q6MKG3	342	38.9	9.28	Uncharacterized protein OS	6.14	3.56	2	2	2	2.03E+08
Bd2463	hypothetical protein	3.47	0.00717	0.75	0.00393	Q6MKD6	186	20.7	7.88	Uncharacterized protein OS	26.88	2.72	3	3	3	4.09E+08
Bd2538	hypothetical protein	4.18	0.00663	3.41	0.0133	Q6MK73	572	63.9	7.59	Uncharacterized protein OS	7.52	4.87	3	3	3	1.37E+08
Bd2553	hypothetical protein	1.33	0.0207	0.21	3.93E-05	Q6MK59	185	20.3	8.05	Uncharacterized protein OS	5.41	1.89	1	1	1	4.12E+08
Bd2850	putative secreted protein	1.57	0.012	0.53	0.00277	Q6MJD0	277	30.4	9.14	Putative secreted protein OS	5.78	0	2	2	2	1.04E+08
Bd2862	hypothetical protein	2.64	1.46E-05	1.23	0.131	Q6MJB9	146	16	7.37	Uncharacterized protein OS	30.14	0	1	1	1	
Bd3137	hypothetical protein	3.23	0.00449	2.41	0.0932	Q6MIL8	1636	181.2	6.38	DUF4953 domain-containing protein OS	1.53	8.3	2	2	3	2.20E+08
Bd3176	hypothetical protein	7.43	0.0115	2.24	0.0581	Q6MII1	271	31	8.6	YkuD domain-containing protein OS	7.75	0	1	1	1	5.92E+07
Bd3279	putative polysaccharide deacetylase	3.63	0.0023	1.31	0.0268	Q6MI90	383	42.8	7.01	Putative polysaccharide deacetylase OS (GlcNAc N deacetylase[14])	7.05	5.07	2	2	2	4.49E+08

Synonym	Gene Name	Fold-Change upon entry to bdelloplast	T-test	Fold-Change upon HI growth	T-test	Accession	# AAs	MW [kDa]	calc. pI	Description	Coverage	Score	# Unique Peptides	# Peptides	# PSMs	Area
Bd3518	hypothetical protein	1.53	0.0179	0.95	0.103	Q6MHM3	147	15.9	4.79	Uncharacterized protein OS	10.88	2	1	1	2	1.26E+08
Bd3618	hypothetical protein	1.89	0.000799	0.84	0.00467	Q6MHD2	257	29.2	6.35	Uncharacterized protein OS	3.89	0	1	1	1	8.30E+08
Bd3701	hypothetical protein	1.86	0.000408	1.74	0.166	Q6MH57	309	34.1	6.14	Uncharacterized protein OS	32.36	30.12	9	9	14	5.02E+09
Bd3702	hypothetical protein	5.08	0.000145	2.29	0.153	Q6MH56	470	50.6	9.14	Uncharacterized protein OS	26.38	16.52	8	8	11	2.11E+09
Bd3748	hypothetical protein	1.51	0.00811	0.52	0.000204	Q6MH17	269	30.4	8.5	Uncharacterized protein OS	5.58	2.81	1	1	1	1.38E+08

Supplementary Figure 1. Representative images of Live/Dead staining at 120 mins post mixing of predator and prey for the single and triple deletion mutant strains. Syto-9 stained (live) cells are false coloured yellow, propidium iodide stained (dead) cells are false coloured magenta. Many such images from three biological repeats were used to derive data on percentage of empty bdelloplasts plotted in figure 1c. Total number of bdelloplasts counted for each strain across entire fields of view from three biological repeats are as follows n=1135 (HD100), n= 1181 (Δ*bd0767)*, n= 1121 (Δ*bd0875)*, n=1223 (Δ*bd3132)*, n=1162 (Δ*Bd0767*Δ*Bd0875*Δ*Bd3132)*. Analysis of *Δbd1483* was performed alongside a *B. bacteriovorus* HD100 control but in separate experiments to analysis of *Δbd0767, Δbd0875, Δbd3132 and Δbd0767Δbd0875Δbd3132*. Total number of bdelloplasts counted for Δ*bd1483* is 1090 alongside HD100 (n=1002). Mean (+SD) percentage empty bdelloplasts for each strain is shown in Supplementary Table 5a. P values from two-tailed unpaired *t*-test are shown in Supplementary Table 5b. Number of bdelloplasts counted across entire fields of view for each biological repeat in shown in Supplementary Table 5c. Scale bar 5 µM. Blue arrows indicate dead (false coloured magenta) empty bdelloplasts observed only in (iii) Δ*bd0875* and (v) *Δbd0767Δbd0875Δbd3132*

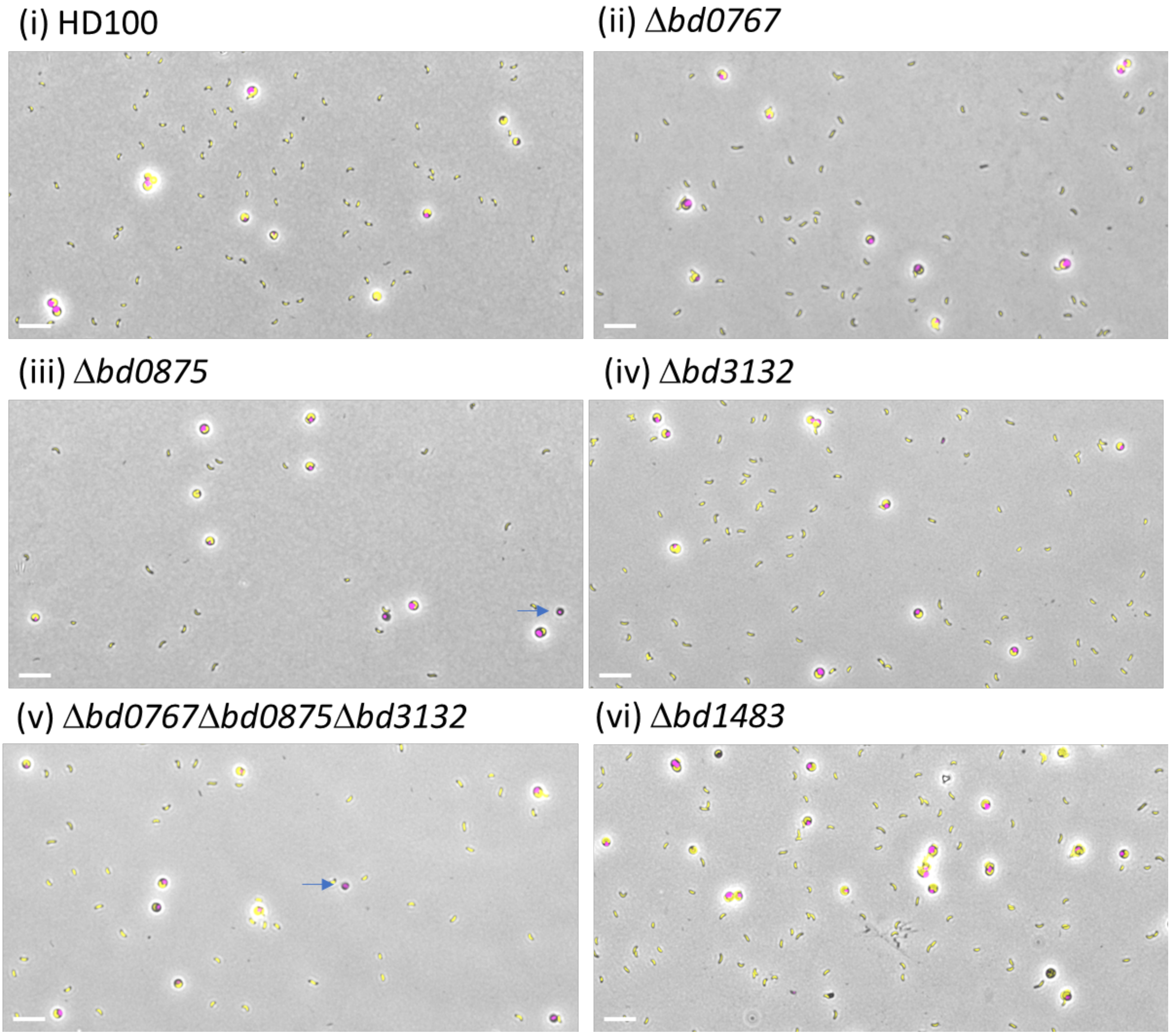

Supplementary Figure 2. Single microscopic examples of predation of *E. coli* S17-1 pZMR100 by *B. bacteriovorus* strains marked with a red Bd0064mCherry cytoplasmic marker: wild type HD100:Bd0064mCherry (i, ii) versus mutant *Δbd0875:*Bd0064mcherry (iii, iv) and complemented strain *Δbd0875:bd0875* Bd0064mcherry (v, vi). Images representative of three biological repeats. Blue arrows denote empty bdelloplasts devoid of an invaded red *B. bacteriovorus* which were only observed in the Δ*bd0875* deletion strain shown here at 120 mins (iii) and 300 mins (iv) post mixing of predator and prey. The phenotype restored to wild type on complementation of the *bd0875* gene (v, vi). Scale bar 5 µM. T 300mins was chosen for the Percoll gradient enrichment, of empty bdelloplasts from attack phase *B. bacteriovor*us, a time where wild type like bdelloplasts were not present (Supplementary Figure 3). Counting the percentage of bdelloplasts devoid of invaded *B. bacteriovorus* used live/dead staining of predation of *E. coli* S17-1 by non-fluorescently-labelled *B. bacteriovorus* strains giving better discrimination between invaded and non-invaded prey (Fig 1c and Supplementary Tables 5d, g and j.)

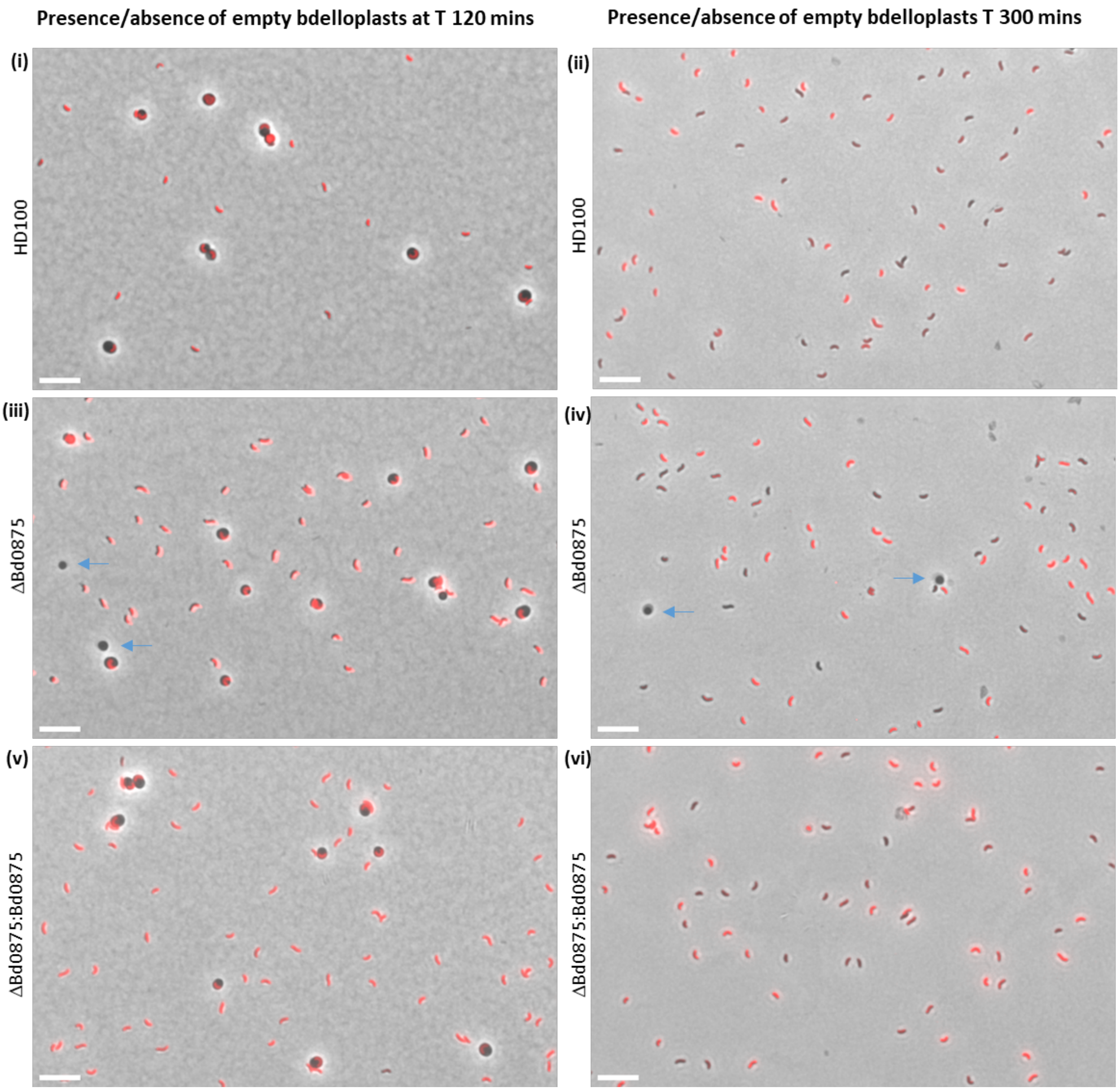

Supplementary Figure 3. Percoll gradient fractions of dead empty prey bdelloplasts (false coloured magenta) from excess attack phase *Bdellovibrio* (false coloured yellow) and prey debris (grey). Live/dead staining was carried out for a sample removed from each of six fractions (i-vi) from Percoll gradient separations. Scale bar 5 µM.

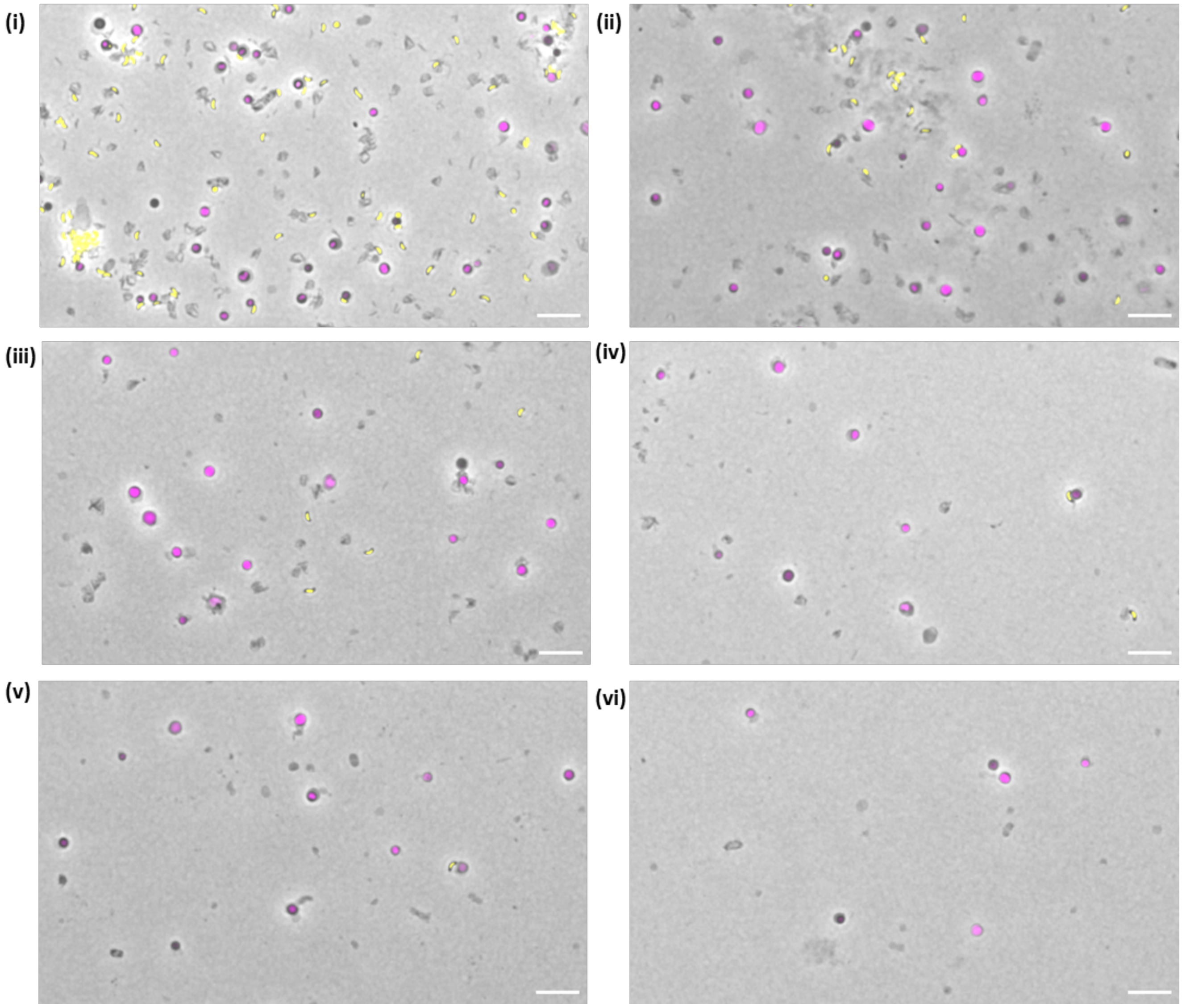

Supplementary Figure 4 Multiple sequence alignment of true Bd0875 homologues. Sequences were identified using the gene cluster feature of KEGG[15] and the subsequent alignment from T-COFFEE[16] were submitted to the ESPript server[17] for graphical annotation of conservation; numbering is with respect to Bd0875 (14-16). The strains used are - Bd0875, *Bdellovibrio bacteriovorus* HD100; Bdt_0838, *Bdellovibrio bacteriovorus* Tiberius; MNR06_08150, *Bdellovibrio reynosensis*; BDW_02990, *Bdellovibrio bacteriovorus* W; DOE51_04220, *Bdellovibrio* sp. NC01; HW988_15020, *Bdellovibrio* sp. KM01; DOM22_15945, *Bdellovibrio* sp. ZAP7; CIK05_03405, *Bdellovibrio* sp. Qaytius; A11Q_762, *Pseudobdellovibrio exovorus*.

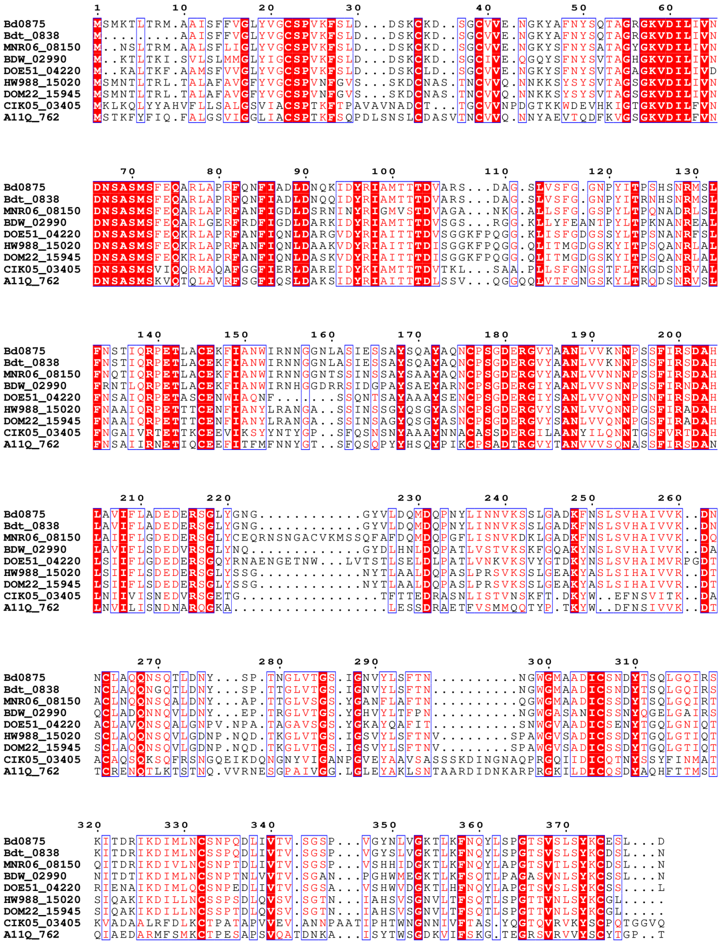

Supplementary References:

1 Lerner, T. R. *et al.* Specialized peptidoglycan hydrolases sculpt the intra-bacterial niche of predatory Bdellovibrio and increase population fitness. *PLoS Pathog* **8**, e1002524, doi:10.1371/journal.ppat.1002524 (2012).

2 Kuru, E. *et al.* Mechanisms of Incorporation for D-Amino Acid Probes That Target Peptidoglycan Biosynthesis. *ACS Chem Biol* **14**, 2745-2756, doi:10.1021/acschembio.9b00664 (2019).

3 Lambert, C., Chang, C.-Y., Capeness, M. J. & Sockett, R. E. The First Bite— Profiling the Predatosome in the Bacterial Pathogen Bdellovibrio. *PLOS ONE* **5**, e8599, doi:10.1371/journal.pone.0008599 (2010).

4 Capeness, M. J. *et al.* Activity of Bdellovibrio hit locus proteins, Bd0108 and Bd0109, links Type IVa pilus extrusion/retraction status to prey-independent growth signalling. *PLoS One* **8**, e79759, doi:10.1371/journal.pone.0079759 (2013).

5 McClure, R. *et al.* Computational analysis of bacterial RNA-Seq data. *Nucleic Acids Res* **41**, e140, doi:10.1093/nar/gkt444 (2013).

6 Schäfer, A. *et al.* Small mobilizable multi-purpose cloning vectors derived from the Escherichia coli plasmids pK18 and pK19: selection of defined deletions in the chromosome of Corynebacterium glutamicum. *Gene* **145**, 69-73, doi:10.1016/0378-1119(94)90324-7 (1994).

7 Fenton, A. K., Kanna, M., Woods, R. D., Aizawa, S. I. & Sockett, R. E. Shadowing the actions of a predator: backlit fluorescent microscopy reveals synchronous nonbinary septation of predatory Bdellovibrio inside prey and exit through discrete bdelloplast pores. *J Bacteriol* **192**, 6329-6335, doi:10.1128/jb.00914-10 (2010).

8 Willis, A. R. *et al.* Injections of Predatory Bacteria Work Alongside Host Immune Cells to Treat Shigella Infection in Zebrafish Larvae. *Curr Biol* **26**, 3343-3351, doi:10.1016/j.cub.2016.09.067 (2016).

9 Hanahan, D. Studies on transformation of Escherichia coli with plasmids. *Journal of molecular biology* **166**, 557-580, doi:10.1016/s0022-2836(83)80284-8 (1983).

10 Rogers, M., Ekaterinaki, N., Nimmo, E. & Sherratt, D. Analysis of Tn7 transposition. *Mol Gen Genet* **205**, 550-556, doi:10.1007/bf00338097 (1986).

11 Rendulic, S. *et al.* A predator unmasked: life cycle of Bdellovibrio bacteriovorus from a genomic perspective. *Science* **303**, 689-692, doi:10.1126/science.1093027 (2004).

12 Caulton, S.G., Lambert, C., Tyson, J. *et al. Bdellovibrio bacteriovorus* uses chimeric fibre proteins to recognize and invade a broad range of bacterial hosts. *Nat Microbiol* **9**, 214–227 (2024). https://doi.org/10.1038/s41564-023-01552-2

13 Ducret, A., Quardokus, E. M. & Brun, Y. V. MicrobeJ, a tool for high throughput bacterial cell detection and quantitative analysis. *Nat Microbiol* **1**, 16077, doi:10.1038/nmicrobiol.2016.77 (2016).

14 Lambert, C. *et al.* Interrupting peptidoglycan deacetylation during Bdellovibrio predator-prey interaction prevents ultimate destruction of prey wall, liberating bacterial-ghosts. *Sci Rep* **6**, 26010, doi:10.1038/srep26010 (2016).

15 Kanehisa, M., Furumichi, M., Sato, Y., Kawashima, M. and Ishiguro-Watanabe, M.; KEGG for taxonomy-based analysis of pathways and genomes. Nucleic Acids Res. 51, D587-D592 doi10.1093/nar/gkac963 (2023)

16 P Di Tommaso, P., Moreti, S., Xenarios, I., Orobitg, M., Montayola, A., Chang, J.M., Taly, J.F., Notredame, C. T-Coffee: a web server for the multiple sequence alignment of protein and RNA sequences using structural information and homology extension doi 10.1093/nar/gkr245 (2011).

17 Robert, X. & Gouet, P. Deciphering key features in protein structures with the new ENDscript server. *Nucleic Acids Res,* 42**,** W320-4 doi 10.1093/Nar/gku316 (2014).
