# Supplementary material for: Prey killing without invasion by Bdellovibrio bacteriovorus defective for a MIDAS-family adhesin
Source: Nat Commun. 2024 Apr 9;15:3078. doi: 10.1038/s41467-024-47412-3 (PMC11003981; doi:10.1038/s41467-024-47412-3)
Supplement: Supplementary file 3 — Description of Additional Supplementary Files [file 41467_2024_47412_MOESM3_ESM.pdf]

Description of Additional Supplementary Files:

Supplementary Dataset 1: Proteomics of predator and prey proteins from empty *E.coli* bdelloplasts preyed upon by Δ*bd0875 B. bacteriovorus*.
