# Supplementary material for: Prey killing without invasion by Bdellovibrio bacteriovorus defective for a MIDAS-family adhesin
Source: Nat Commun. 2024 Apr 9;15:3078. doi: 10.1038/s41467-024-47412-3 (PMC11003981; doi:10.1038/s41467-024-47412-3)
Supplement: Supplementary file 5 — Reporting Summary [file 41467_2024_47412_MOESM5_ESM.pdf]

nature portfolio

Corresponding author(s): Andrew Lee Lovering and Renee Elizabeth Sockett

Last updated by author(s): 2024/03/19

Reporting Summary

Nature Portfolio wishes to improve the reproducibility of the work that we publish. This form provides structure for consistency and transparency in reporting. For further information on Nature Portfolio policies, see our Editorial Policies and the Editorial Policy Checklist.

Please do not complete any field with "not applicable" or n/a. Refer to the help text for what text to use if an item is not relevant to your study.
For final submission: please carefully check your responses for accuracy; you will not be able to make changes later.

Statistics

For all statistical analyses, confirm that the following items are present in the figure legend, table legend, main text, or Methods section.

n/a	Confirmed	
☐	☒	The exact sample size (n) for each experimental group/condition, given as a discrete number and unit of measurement
☐	☒	A statement on whether measurements were taken from distinct samples or whether the same sample was measured repeatedly
☐	☒	The statistical test(s) used AND whether they are one- or two-sided<br>*Only common tests should be described solely by name; describe more complex techniques in the Methods section.*
☒	☐	A description of all covariates tested
☐	☒	A description of any assumptions or corrections, such as tests of normality and adjustment for multiple comparisons
☐	☒	A full description of the statistical parameters including central tendency (e.g. means) or other basic estimates (e.g. regression coefficient) AND variation (e.g. standard deviation) or associated estimates of uncertainty (e.g. confidence intervals)
☐	☒	For null hypothesis testing, the test statistic (e.g. F, t, r) with confidence intervals, effect sizes, degrees of freedom and P value noted<br>*Give P values as exact values whenever suitable.*
☒	☐	For Bayesian analysis, information on the choice of priors and Markov chain Monte Carlo settings
☒	☐	For hierarchical and complex designs, identification of the appropriate level for tests and full reporting of outcomes
☒	☐	Estimates of effect sizes (e.g. Cohen's d, Pearson's r), indicating how they were calculated

Our web collection on statistics for biologists contains articles on many of the points above.

Software and code

Policy information about availability of computer code

Data collection	Images were acquired with Nikon NIS Elements software Version AR 5.11.02 64 bit. The AlphaFold models of Bd0875 and Bd1483 proteins were from DB version 2022-11-01, created with the AlphaFold Monomer v2.0 pipeline (accessed at https://alphafold.ebi.ac.uk/entry/Q6MPH9 and https://alphafold.ebi.ac.uk/entry/Q6MMY6, respectively). Sequences for protein alignments were acquired from KEGG Kanehisa, M., Furumichi, M., Sato, Y., Kawashima, M. and Ishiguro-Watanabe, M.; KEGG for taxonomy-based analysis of pathways and genomes. Nucleic Acids Res. 51, D587-D592 (2023).
Data analysis	Image analysis was carried out with Fiji Version 1.52n, Microbe J version 5.13j, Graph Pad Prism 8.0 Excel 2016. Protein Sequence alignments were made with PROMALS3D - Pei, J., Kim, B. H. & Grishin, N. V. PROMALS3D: a tool for multiple protein sequence and structure alignments. Nucleic Acids Res 36, 2295-2300, doi:10.1093/nar/gkn072 (2008). and TCoffee- TCOFFEE-https://<br>eu ropepmc.org/a rticle/M ED/35412617<br>and displayed with ESPRIPT - Robert, X. & Gouet, P. Deciphering key features in protein structures with the new ENDscript server. Nucleic Acids Res 42, W320-324, doi:10.1093/nar/gku316 (2014).

For manuscripts utilizing custom algorithms or software that are central to the research but not yet described in published literature, software must be made available to editors and reviewers. We strongly encourage code deposition in a community repository (e.g. GitHub). See the Nature Portfolio guidelines for submitting code & software for further information.

Data

Policy information about availability of data

All manuscripts must include a data availability statement. This statement should provide the following information, where applicable:

- Accession codes, unique identifiers, or web links for publicly available datasets
- A description of any restrictions on data availability
- For clinical datasets or third party data, please ensure that the statement adheres to our policy

Source data for the Figures and statistical analyses are presented in a Source data Excel file called Source data for Tyson et al 2024, accompanying this paper.
Transcriptomic data for B. bacteriovorus bd1291 deletion mutant and wild type HD100 strain are deposited at the (Genbank) Sequence Read Archive (SRA) https://www.ncbi.nlm.nih.gov/sra/docs/
and approved with accession numbers SAMN38260227- SAMN38260240. https://www.ncbi.nlm.nih.gov/sra/?term=SAMN38260227 - https://www.ncbi.nlm.nih.gov/sra/?term=SAMN38260240
The previously published transcriptional data (REF13) of wild type predatory B. bacteriovorus HD100 upon invasion of prey, used to compare to the proteomics in Supplementary Dataset 1 are available at GEO Gene expression Omnibus GSE9269. [https://www.ncbi.nlm.nih.gov/geo/query/acc.cgi?acc=GSE9269]

The AlphaFold models of Bd0875 and Bd1483 proteins were from DB version 2022-11-01, created with the AlphaFold Monomer v2.0 pipeline (accessed at https://alphafold.ebi.ac.uk/entry/Q6MPH9 and https://alphafold.ebi.ac.uk/entry/Q6MMY6, respectively).

Proteomic Data
The mass spectrometry proteomics data have been deposited to the ProteomeXchange Consortium via the PRIDE29 partner repository with the dataset identifier PXD050423. https://www.ebi.ac.uk/pride/archive/projects/PXD050423
They are also presented in Supplementary Dataset 1 of this paper.

Research involving human participants, their data, or biological material

Policy information about studies with human participants or human data. See also policy information about sex, gender (identity/presentation), and sexual orientation and race, ethnicity and racism.

Reporting on sex and gender	N/A for our bacterial experimental work.
Reporting on race, ethnicity, or other socially relevant groupings	N/A for our bacterial experimental work.
Population characteristics	N/A for our bacterial experimental work.
Recruitment	N/A for our bacterial experimental work.
Ethics oversight	N/A for our bacterial experimental work.

Note that full information on the approval of the study protocol must also be provided in the manuscript.

Field-specific reporting

Please select the one below that is the best fit for your research. If you are not sure, read the appropriate sections before making your selection.

☒ Life sciences ☐ Behavioural & social sciences ☐ Ecological, evolutionary & environmental sciences

For a reference copy of the document with all sections, see nature.com/documents/nr-reporting-summary-flat.pdf

Life sciences study design

All studies must disclose on these points even when the disclosure is negative.

Sample size	No calculation was performed to predetermine sample size. We chose sample sizes based on methods that have previously produced reliable and statistically robust data in this bacterial system (Harding C et al Nature Comms 2020, Lerner TR et al 2012 PLoS Pathogens, Kuru E. et al Nat Micro 2017). Exact sample sizes as n= are reported for every experiment in the relevant Figures and legends.
Data exclusions	No data were excluded from analysis
Replication	Three independent biological repeats were performed for the microscopic phenotypic analyses with each repeat consistently reproducing the findings and this is described in figure legends 1a-1c and in data analysis in methods, the Source data file and Supporting information . In Figure 1d and Figure 1e two different staining methods were used to illustrate the diverse positions of the 875 protein . This diversity was described qualitatively in the paper and across Figure 1d (2 replicates)and Figure 1e (1 replicate) images from a total of three replicates were carried out as noted in the legends. For the Proteomic analysis a single replicate of the experiment was carried out as described in the

manuscript. For the pilot bd1291 transcriptomics which generated testable candidates for the study a single replicate of the experiment was carried out as explained in the paper. Then the resulting candidate genes were tested by gene deletion, leading to the discovery of the Bd0875 gene phenotype.

Randomization	For each biological repeat experiment separate cultures of the bacteria were raised and colonies or plaques of the prey or predator bacteria were chosen at random for onward culturing for pheotyping assays. No covariates were studied so this part isnt relevant.
Blinding	Not relevant as no group allocation was made in our study.

Reporting for specific materials, systems and methods

We require information from authors about some types of materials, experimental systems and methods used in many studies. Here, indicate whether each material, system or method listed is relevant to your study. If you are not sure if a list item applies to your research, read the appropriate section before selecting a response.

Materials & experimental systems

n/a	Involved in the study	
☐	☒	Antibodies
☒	☐	Eukaryotic cell lines
☒	☐	Palaeontology and archaeology
☒	☐	Animals and other organisms
☒	☐	Clinical data
☒	☐	Dual use research of concern
☒	☐	Plants

Methods

n/a	Involved in the study	
☒	☐	ChIP-seq
☒	☐	Flow cytometry
☒	☐	MRI-based neuroimaging

Antibodies

Antibodies used	All from Thermofisher: rabbit anti-mCherry polyclonal antibody Catalog number PAS-34974 used at 1:1000 dilution<br>Goat-anti-rabbit lgG-H-L-Highly-Cross-Adsorbed-Secondary-Antibody-Polyclonal Alexa Fluor Plus 488 Catalog number A32731 used at 1:1000 dilution or Goat-anti-rabbit lgG-H-L-Highly-Cross-Adsorbed-Secondary-Antibody-Polyclonal Alexa Fluor Plus 555 Catalog number<br>A32732 used at 1:1000 dilution
Validation	Publications for each antibody at ThermoFisher website https://www.thermofisher.com/antibody/product/mCherry-Antibody-Polyclonal/<br>PAS-34974 https://www.thermofisher.com/antibody/product/Goat-anti-Rabbit-IgG-H-L-Highly-Cross-Adsorbed-Secondary-Antibody-Polyclonal/A32731https://www.thermofisher.com/antibody/product/Goat-anti-Rabbit-IgG-H-L-Highly-Cross-Adsorbed-Secondary-Antibody-PolN/A yclonal/A32732.

Plants

Seed stocks	none used
Novel plant genotypes	N/A
Authentication	N/A

This checklist template is licensed under a Creative Commons Attribution 4.0 International License, which permits use, sharing, adaptation, distribution and reproduction in any medium or format, as long as you give appropriate credit to the original author(s) and the source, provide a link to the Creative Commons license, and indicate if changes were made. The images or other third party material in this article are included in the article's Creative Commons license, unless indicated otherwise in a credit line to the material. If material is not included in the article's Creative Commons license and your intended use is not permitted by statutory regulation or exceeds the permitted use, you will need to obtain permission directly from the copyright holder. To view a copy of this license, visit http://creativecommons.org/licenses/by/4.0/
